# A Report of Two Interesting Cases

**Published:** 1899-08

**Authors:** Marcus F. Carson

**Affiliations:** Griffin, Ga.


### A REPORT OF TWO INTERESTING CASES.

By MARCUS F. CARSON, M.D.,
Griffin, Ga.

I wish to treat first of a very simple but interesting case of pleurisy in a man, aged 40, whose occupation is that of a cotton mill hand.

Two months ago, after coming out of a warm room, he caught cold, and soon after, noticed a severe pain under cardiac region; he continued at work, but three weeks later—that is, after first attack—he noticed that his pain suddenly became very much worse, and was aggravated by coughing. He went to bed, but did not consult a physician. A week ago he felt improved and returned to work, but the pain returned, his respiration hurried and very painful. He then consulted me, when I found the entire left side of his chest completely dull. I could hear no breath-sounds, but there was a whispering pectoriloquy at one point.

When I placed patient flat upon his back the cardiac dullness was clearly on the right side of sternum, and the cardiac impulse could be felt in the fourth right interspace, internal to the nipple line. I could also hear friction sounds at the right base. In fact there was an enormous effusion displacing the heart to the right side.

This patient has had several ounces drawn off, but the effusion still continues, and the chest remains dull to the clavicle and to the middle line.

Now, on examining the heart, I find it gradually returning to its normal position, but can detect no apex, which is not unusual with a heart that has been driven over to the other side, for on its return it gets behind the sternum. Movement on left side is very much restricted. No breath-sounds can be heard below the level of the second interspace, and the voice is only heard through the ear and not the stethoscope. There is a slight bronchophony, which I think is due to the solidity of the lung behind the fluid.

Acute pleurisy is usually divided into acute dry pleurisy and acute pleurisy with effusion. I don't believe that there is practically any difference between the acute dry and the subacute pleurisy, and as a rule the temperature in this form does not range so high as in the effusive variety. In the study of the latter variety we have some points of very great interest; for example, we may have acute pleurisy coming on idiopathically from a chill, and second, cases as the result of acute febrile diseases, as rheumatic fever.

I am thoroughly convinced that a great many of the simply-caught-cold pleurisies are of a tubercular origin, and that we should always be on our guard in our prognosis of these cases. Our inability to find the bacillus does not stand for much, for in many undoubted tubercular diseases you may search diligently without discovering the bacillus.

In the past eighteen months I have examined specimens from five patients of the ordinary "caught-cold variety" of pleurisy, finding the bacillus in only two of them; but because I failed to find the bacillus in three of my cases was no reason why I should not be suspicious of tuberculosis. Since that time two of my cases in which I did not discover the bacillus have died of tuberculosis, while only one of the cases in which I was able to detect the germ has died.

I had infinitely rather treat a case of pleurisy the result of an acute febrile disease, than one of the "caught-cold-chill" variety.

I do not believe that all these cases are tubercular, but the history of all my cases shows that if the original disease was not tubercular, tubercle certainly became an element in their later history.

I think these facts should make us careful in giving a prognosis in cases of acute pleurisy without any known cause but a chill. A patient suffering from acute pleurisy may have a better chance of recovery than a subject of acute croupous pneumonia, but you will find that the pneumonia patient will not have the slightest trouble afterwards, while the pleurisy patient, in many cases, will die of tuberculosis.

My second case is interesting to contrast with the case I have just described. A lady thirty years of age had pleurisy four years ago. The right side of the chest is much larger than the left. Loss of movement is obvious in both cases. In the former the lung is partly crushed. In this case the loss of movement is due to the strapping. There is no fluid in the chest of this lady at present, and from the history she has had a "caught-cold" pleurisy, and there is undoubtedly evidence to show that she is becoming tuberculous, probably caused by the fluid being allowed to remain in her chest too long when she was first attacked, crippling the lung. One peculiar symptom in this case is a violent attack of herpes zoster, which I am unable to account for, unless it is due to the compression of one of the intercostal nerves, or internal pressure upon the ganglion by hemorrhage. Patient was confined to her bed for three months, when her chest was tapped. On examination I found very little movement, and on percussion of back there is complete dullness. There was definite bronchophony, so much that I first thought it a very large cavity. I also found signs of softening and fine crepitations. The expectoration was not purulent.

I think these two are very analogous cases. They are both left-sided pleurisies. In the man the chest is full of fluid on one side, the pleuræ roughened, the heart pushed to the right side, and lung almost completely collapsed; there is no high-pitched note at the apex showing the compensatory emphysema which is common in pleurisy, I think, for the simple reason that the effusion is so high that it covers the apex as well as the rest of the lung, leaving no room for emphysema.

As to the treatment of acute pleurisy there are very few points, but they are very important. I believe that the pain should be controlled by opium.

The cough is nervous and the pain is very acute. Opium quiets the heart, relieves the pain, without checking the large quantity of secretion.

As to the effusion, there is no doubt that the early drawing off of the pleuritic effusion, and seeing that a large effusion that is crushing the lung does not remain too long, is one of the chief points in the treatment of pleurisy, because if the lung is compressed too long, as in the first case referred to, it loses its elasticity, becomes enormously congested, the circulation through it is impeded, all the capillaries and small arteries are distorted, and as a result the entire lung atrophies.

One important point in aspirating with little pressure is, the fluid may cease to flow through the needle before the liquid has all come away, for the simple reason that the negative pressure in the chest equals that on the outside. Our text-books teach us that we should not draw off the fluid until the temperature is normal, and that if the case be tubercular, we should never draw it off. Both of these injunctions are incorrect. We do draw off the fluid if it is embarrassing, whatever the circumstances or whatever the temperature may be. As to the amount that should be drawn off, there is no fixed rule. I think the main point is to judge by the pulse and the presence of cough. When the patient begins to cough I always withdraw the aspirator. I believe in controlling the quantity of fluid, giving as little as possible.

I am satisfied that both of these cases are going to terminate in fibroid phthisis.

